# Sauchinone controls hepatic cholesterol homeostasis by the negative regulation of PCSK9 transcriptional network

**DOI:** 10.1038/s41598-018-24935-6

**Published:** 2018-04-30

**Authors:** Hee-Sung Chae, Byoung Hoon You, Dong-Yeop Kim, Hankyu Lee, Hyuk Wan Ko, Hyun-Jeong Ko, Young Hee Choi, Sun Shim Choi, Young-Won Chin

**Affiliations:** 1 College of Pharmacy and Integrated Research Institute for Drug Development, Dongguk University-Seoul, Goyang, Gyeonggi-do 10326 Republic of Korea; 2 Division of Biomedical Convergence, College of Biomedical Science, and Institute of Bioscience & Biotechnology, Kangwon National University, Chuncheon, Gangwon 24341 Republic of Korea; 3 Laboratory of Microbiology and Immunology, College of Pharmacy, Kangwon National University, Chuncheon, Gangwon 24341 Republic of Korea

## Abstract

Whole-transcriptome analysis and western blotting of sauchinone-treated HepG2 cells demonstrated that sauchinone regulated genes relevant to cholesterol metabolism and synthesis. In particular, it was found that the expression of proprotein convertase subtilisin/kexin type 9 (PCSK9) was downregulated, and the expression of low density lipoprotein receptor (LDLR) was upregulated in sauchinone-treated HepG2 cells. Consequently, LDL-cholesterol (LDL-C) uptake was increased. As a transcriptional regulator of PCSK9 expression, sterol regulatory elements binding protein-2 (SREBP-2) was proposed by transcriptome analysis and western blotting. Oral administration of sauchinone increased hepatic LDLR through PCSK9 inhibition in obese mice and showed the reduced serum LDL-C levels and downstream targets of SREBP-2. Thus, it is evident that sauchinone reduces hepatic steatosis by downregulating the expression of hepatic PCSK9 via SREBP-2.

## Introduction

Cholesterol homeostasis is regulated by a family of transcription factors called sterol regulatory elements binding proteins (SREBPs)^1^. SREBPs directly activate the expression of genes involved in synthesis and metabolism of cholesterol, fatty acids, and triglyceride (TG)^2^. The SREBP-1c isoform activates all lipogenic genes in the liver. SREBP-1c has been implicated in the development of hepatic steatosis^3^. SREBP-2 is involved in regulating cholesterol synthesis and metabolism genes such as proprotein convertase subtilisin/kexin type 9 (PCSK9), hydroxy-3-methylglutaryl-coenzyme A reductase (HMGCR), cholesteryl ester transfer protein (CETP), and squalene epoxidase (SQLE)^4–6^. A SQLE inhibitor was expected to be a candidate in the treatment of hypercholesterolemia^7^. SREBP-2 activates cholesterol synthesis by upregulating the expression of HMGCR.

PCSK9 is a secreted protein that is avidly expressed in the liver and which contributes to cholesterol homeostasis^8^. Some patients with low levels of proprotein convertase subtilisin/kexin type 9 (LDL-C) harbor PCSK9 loss-of-function mutations; these patients have a reduced incidence of coronary heart disease^9–11^. PCSK9 binds to the epidermal growth factor (EGF), a domain of the LDL receptor (LDLR)^12^. PCSK9 promotes degradation of these cell surface LDL receptors in selected cell types^13^. Statins such as simvastatin are clinically used by reducing the quantity of LDL-C^14,15^.

Despite the efficacy of statin therapy, some patients with familial hypercholesterolemia, an inherited autosomal dominant disorder characterized by extremely high levels of LDL-C still face substantial residual risk associated with high levels of LDL-C since statin therapy is not completely successful in lowering LDL-C levels^16,17^. High expression of PCSK9 has been observed, particularly in untreated heterozygous or homozygous familial hypercholesterolemia patients. In one study, high-dose statin therapy lowered LDL-C concentrations in both groups of patients as expected, and simultaneously elevated PCSK9 levels^18^. PCSK9 inhibition might be an alternative monotherapy for hypercholesterolemic patients who cannot tolerate statins^19^. Hence, treatment with antibody-based drug candidates in the absence or presence of statins targets PCSK9 activity to accomplish LDL-C control^20^. Inhibition of PCSK9 has emerged as an attractive target to control LDL-C levels^21^. Two antibody-based drugs that directly target PCSK9 were approved by United State Food and Drug Administration (USFDA) in 2015 and several more candidates are undergoing clinical trials^22,23^. So far, there are only a few reports regarding natural products with PCSK9 inhibitory activity^24,25^.

Sauchinone isolated from *Saururus chinensis* has hepatoprotective, anti-inflammatory, and anti-steatosis activity^26–31^. We previously reported that sauchinone was distributed in relatively high levels in the liver^32^. Also, sauchinone inhibits hepatic steatosis mediated by the SREBP isoform, SREBP-1c, and activates AMP-activated protein kinase (AMPK)^31^. The responsible genes, such as the gene encoding PCSK9, have not been determined.

In the present study, hepatic cholesterol homeostasis by sauchinone was analyzed by entire mRNA sequencing (whole-transcriptome analysis) *in vitro* and the correlated genes were proposed. We also assessed the regulation of hepatic steatosis by sauchinone *in vivo* and the proposed genes including PCSK9 and SREBP-2.

## Results

### Sauchinone inhibits lipid metabolic pathways

**Figure 1:**
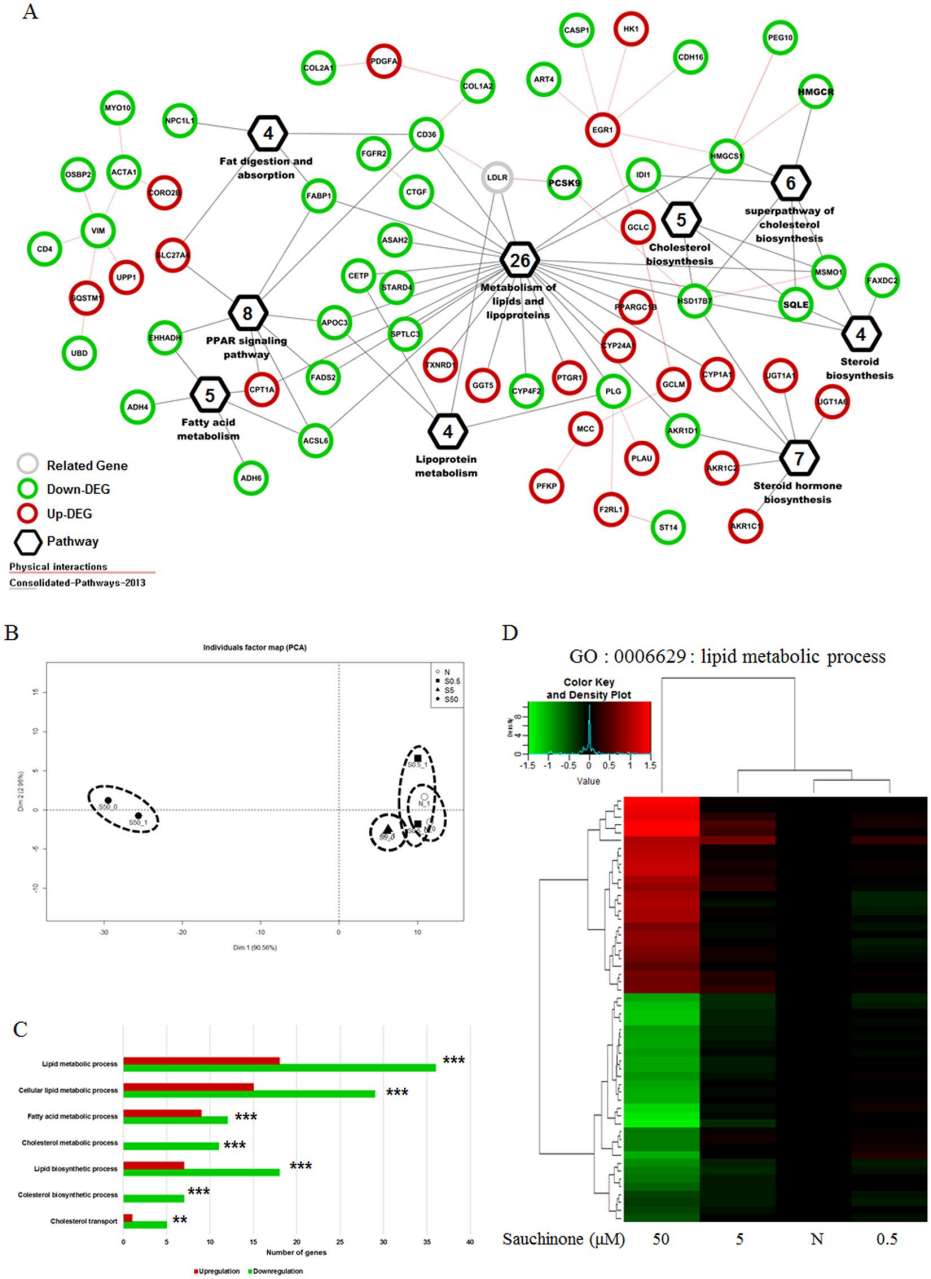
Metabolic gene profiling of HepG2 cells treated with sauchinone for 24 hours. (**A**) The protein-protein interaction network for the 280 DEGs was analyzed with GeneMANIA (ver. 3.4.1) performed with the Cytoscape plugin of the Cytoscape (ver. 3.5.1, http://www.cytoscape.org/) network visualization and analysis environment. ‘Physical interaction’ was chosen with the default options, and the ‘2013 consolidated-pathway’ option was selected to find associations among the 281 DEGs. (**B**) We used R (ver. 3.3.2) for all statistical analysis (https://cran.r-project.org/). The ‘factoMineR’ (http://factominer.free.fr) and ‘rgl’ (https://r-forge.r-project.org/projects/rgl/) packages were used for principal component analysis (PCA) and visualization. (**C**) Enrichment of KEGG pathways in the metabolic gene profiling analysis was determined using the DAVID software. Benjamini-Hochberg adjusted p values are shown for each indicated bar (***p < 0.0001, **p < 0.05, *p < 0.1). (**D**) Hierarchical clustering of all metabolic genes regulated by acRoots in LM3 cells. The genes are annotated to the term “metabolic process” (GO: 0008152) or its children in Gene Ontology.

To assess the effects of sauchinone on HepG2 cells, the RNA-seq approach was used for the mRNAs collected from HepG2 cells treated with various doses of sauchinone. We identified a total of 280 differentially expressed genes (DEGs) that showed at least a two-fold change in expression of the case group treated with sauchinone compared to the untreated control group (Fig. 1A, Supplementary Table S1). Genes involved in lipid metabolic process, cholesterol biosynthetic process, and peroxisome proliferator-activated receptor (PPAR) signaling were significantly modulated by sauchinone (Supplementary Tables S2 and S3). Particularly, genes categorized into gene ontology (GO) terms of “lipid metabolic process” (GO:0006629), and “cholesterol metabolic process” (GO:0008203) were enriched in the DEGs (http://amigo1.geneontology.org/) (Supplementary Table S2). These genes were shown to be connected with each other functionally in a protein-protein interaction network (Fig. 1A). A principal component analysis (PCA) plot using the 280 DEGs showed a clear differentiation among HepG2 cells treated with different doses of sauchinone. Heat mapping also confirmed the dose-dependent regulation of sauchinone in the gene expressions (Fig. 1C). Interestingly, down-regulated genes were present in a significantly higher amount than up-regulated genes in the sauchinone-regulated lipid metabolism genes (Fig. 1D). The results implicate sauchinone in the alteration of lipoprotein metabolism in HepG2 cells.

### Sauchinone regulates genes involved in the lipid metabolism pathway

**Figure 2:**
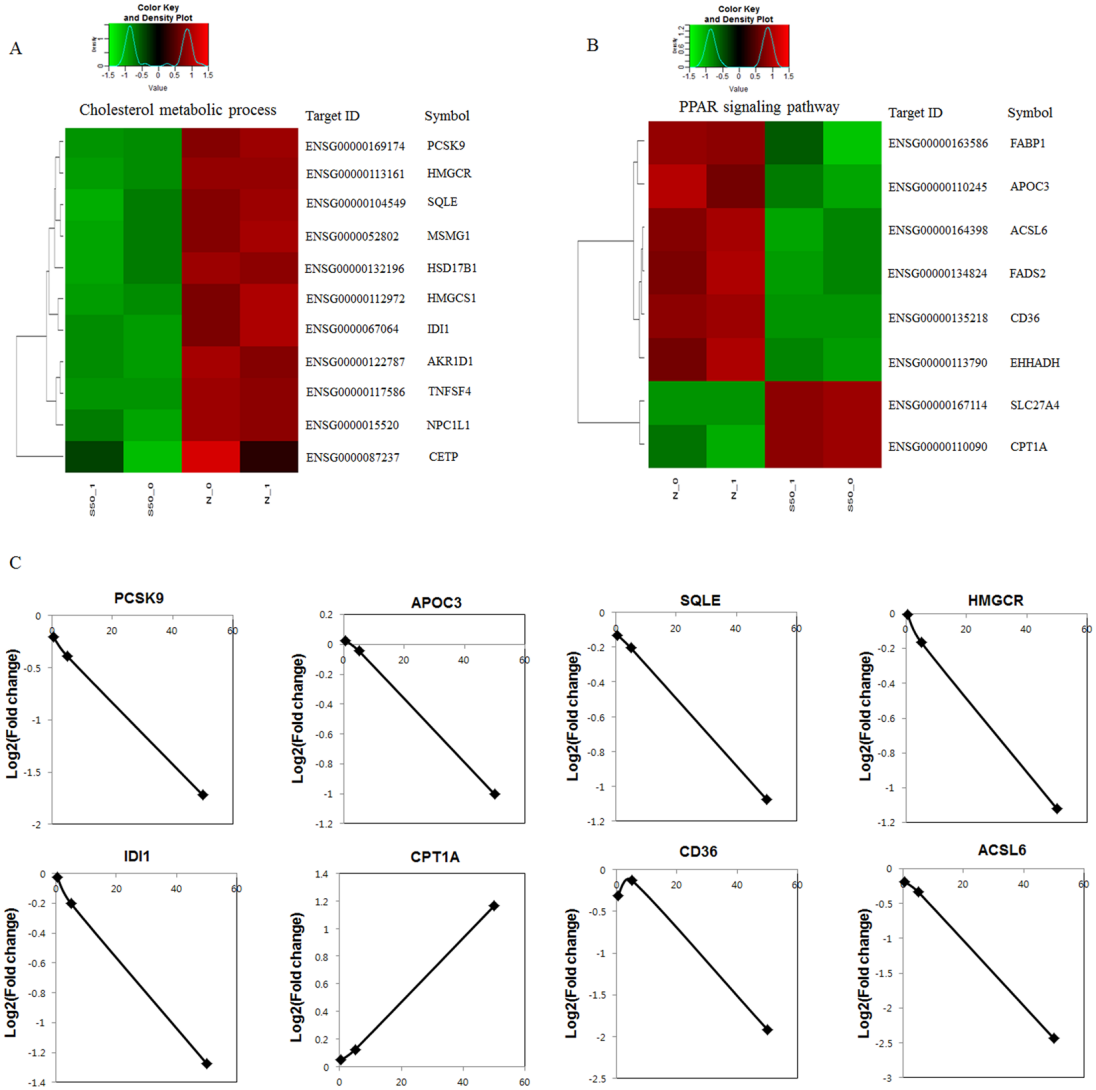
Metabolic genes regulated in HepG2 cells by sauchinone. (**A**) Sauchinone profiles for Cholesterol metabolic process: eleven genes for which the mRNA sequencing was >2.0 and the flag was the p value (representing a higher expression). (**B**) Sauchinone profiles for PPAR signaling pathway: eight genes for which the mRNA sequencing was >2.0 and the flag was the p value (representing a higher expression). (**C**) Validation of the metabolic gene profiles for the example genes by mRNA sequencing in HepG2 cells treated with sauchinone in a dose-dependent manner. Data represent the mean ± SD of triplicate samples.

To investigate cholesterol homeostasis signaling that affects cholesterol metabolism, DEGs were compared in non-treated (control), and sauchinone-treated HepG2 cells. These DEGs fell into two additional categories: 11 and 8 genes regulated in sauchinone-treated cells were involved with cholesterol metabolism and PPAR signaling, respectively. Pathway analysis revealed that PCSK9, apolipoprotein C3 (APOC3), squalene epoxidase (SQLE), HMGCR, isopentenyl-diphosphate delta isomerase 1 (IDI1), carnitine palmitoyltransferase 1 (CPT1A), cluster of differentiation 36 (CD36) and acyl-CoA synthetase long-chain family member 6 (ACSL6) were the central genes in the metabolism of lipids and lipoproteins, with the assumption that sauchinone regulates cholesterol by affecting PCSK9. We selected five genes from each of the two clusters that had a signal >6.0 in the mRNA. The q value (representing a higher expression) was used to validate by quantitative reverse transcription PCR (qRT-PCR) (Fig. 2). The results were consistent with those of the qRT-PCR mRNA expressions, which validated the accuracy of the mRNA sequencing. Interestingly, PCSK9 was the metabolic gene that was the significantly down-regulated by sauchinone in HepG2 cells (Fig. 2B and C).

### Sauchinone regulates cholesterol signaling in HepG2 cells

**Figure 3:**
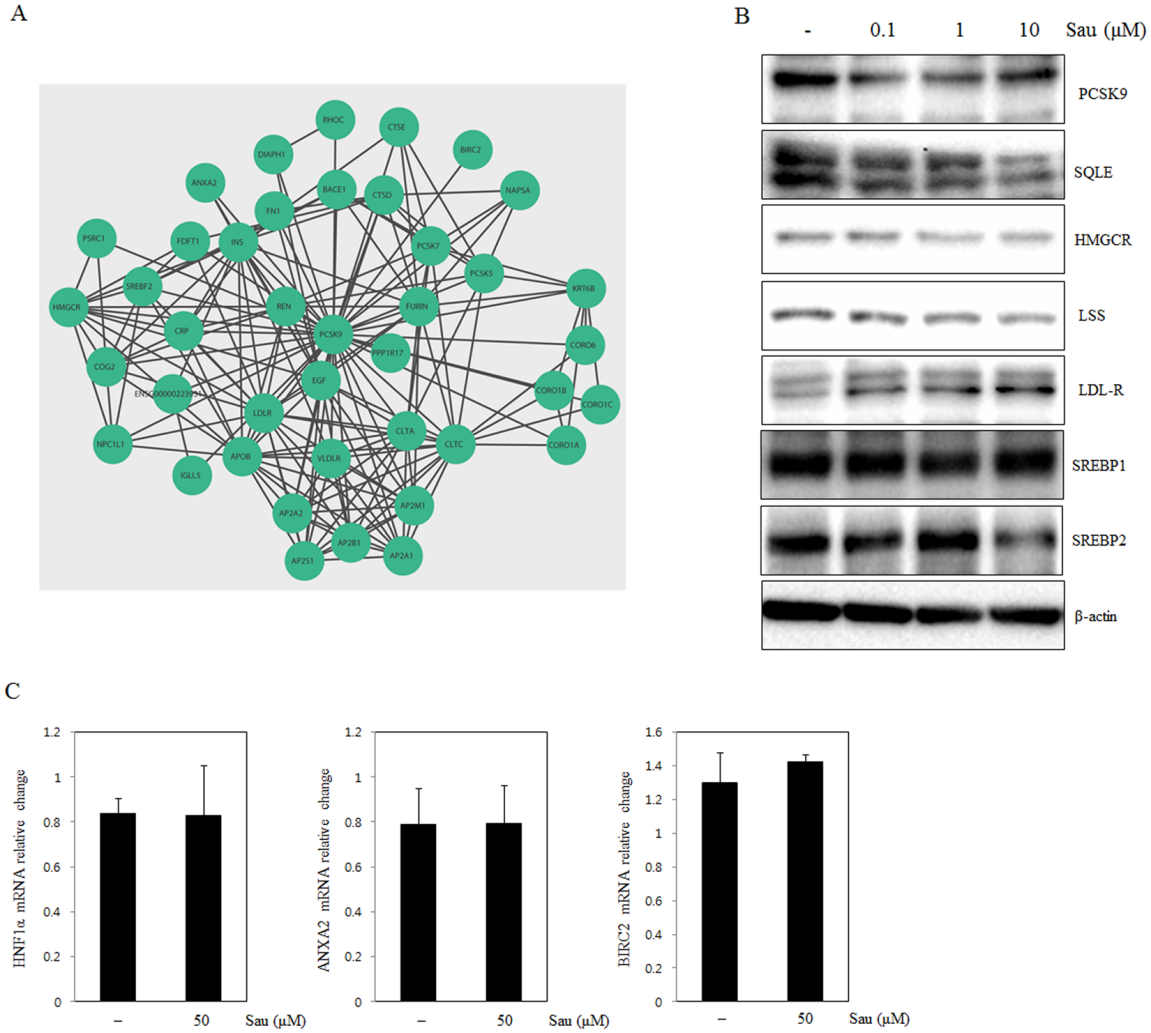
Modulation of the cholesterol signaling pathway in HepG2 cells treated with sauchinone. (**A**) PCSK9 molecular network, obtained with Cytoscape v. 3.5.1^56^ using STRING database^57^. (**B**) PCSK9 signaling pathway and associated changes assessed by western blot analysis. (**C**) Genes known to interact with PCSK9 such as HNF1α, ANXA2 and BIRC2 change in mRNA levels assessed by qRT-PCR analysis.

PCSK9 is a proprotein convertase that regulates the post-transcriptional degradation of the LDLR to reduce cholesterol uptake^33^. To determine whether sauchinone regulats cholesterol metabolism in HepG2 cells in a PCSK9-dependent process, we analyzed the molecular network (www.string-db.org) and signaling pathways associated with PCSK9 based on previous reports. We selected the interactions whose integrated scores were >0.4 (the default threshold in the STRING database) to construct the PCSK9 network. Finally, the obtained PCSK9 networks were visualized using Cytoscape ver.3.5.1 software (Fig. 3A)^34^. The corresponding changes in the mRNA level are shown next to the gene symbols. Molecular network analysis indicated that sterol regulatory element binding transcription factor 2 (SREBF2) and LDLR were closely linked to PCSK9. mRNA sequencing data revealed that, LDLR was not significantly changed by sauchinone, while LDLR protein was increased by sauchinone (Fig. 3B). Thus, the increase of LDLR by sauchinone was due to down-regulation of PCSK9. Signaling pathway analysis revealed that SQLE, LDLR and HMGCR, which are involved in cholesterol uptake and synthesis, respectively, were down-regulated, suggesting the possibility that sauchinone inhibits cholesterol synthesis by altering SQLE, and HMGCR expression in HepG2 cells.

### Sauchinone inhibits PCSK9 expression and regulates cholesterol metabolism

**Figure 4:**
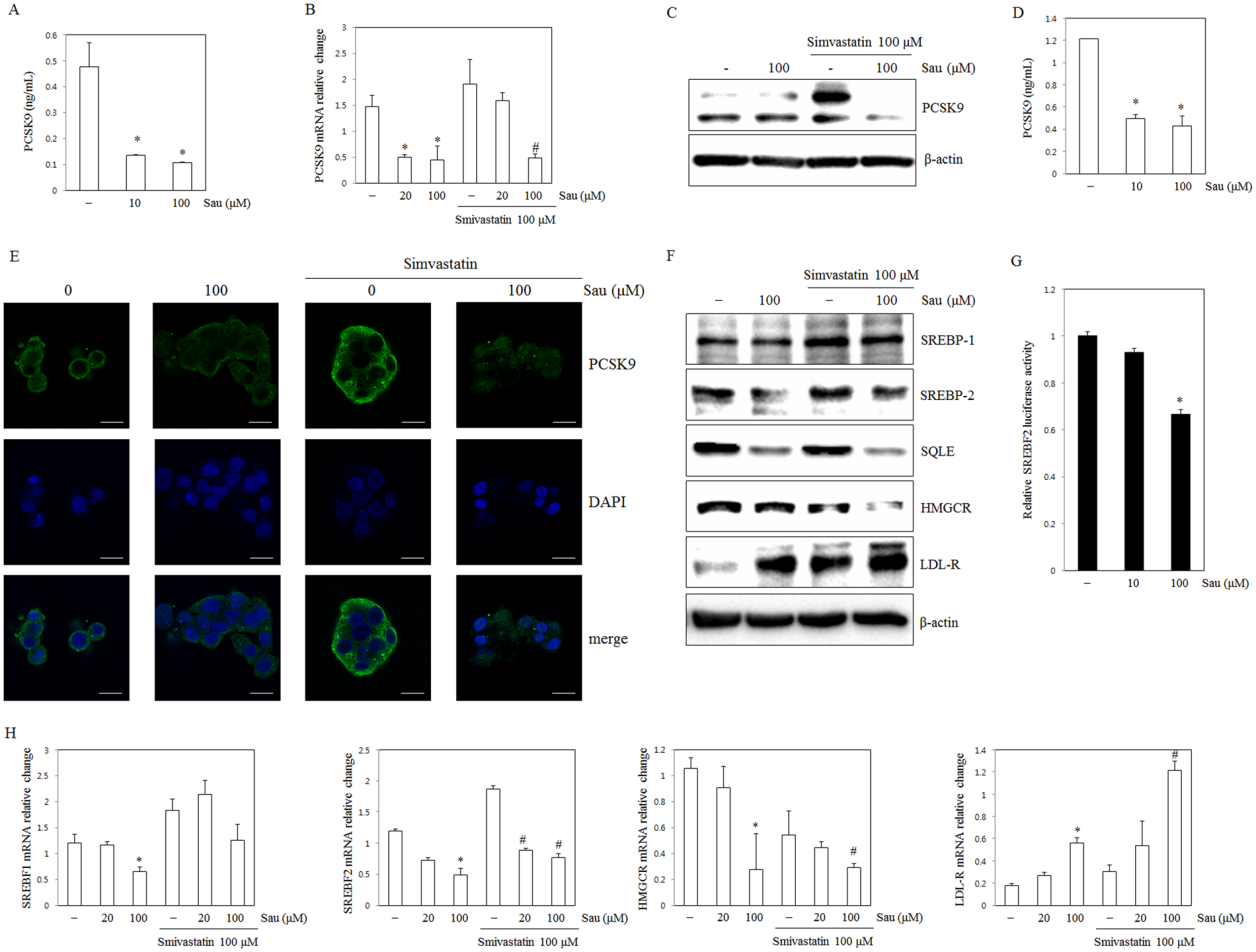
Effect of sauchinone on PCSK9 inhibition in the HepG2 human hepatocellular liver carcinoma cell line. (**A**) The expression of PCSK9 was assayed by ELISA in HepG2 cells treated with sauchinone (10, and 100 μM) for 24 hours. (**B**) The mRNA expression of PCSK9 was assayed by qRT-PCR in cells treated with sauchinone (20, and 100 μM), and simvastatin (100 μM) for 24 hours. (**C**) The protein expression of PCSK9 was assayed by Western blotting in cells treated with sauchinone (100 μM) and simvastatin (100 μM) for 24 hours. (**D**) The expression of PCSK9 was assayed by ELISA in mouse primary hepatocytes treated with sauchinone (10, and 100 μM) for 24 hours. (**E**) Expression of PCSK9 by sauchinone. Cells were cultured for 24 hours, fixed, permeabilized, and incubated with a rabbit monoclonal anti- PCSK9 antibody followed by an Alexa 488 conjugated anti-rabbit IgG (green). The nuclei of the corresponding cells were visualized by 4′,6-diamidino-2-phenylindole (DAPI) staining (blue). (**F**) The protein expression of SREBP1 SREBP2, SQLE, HMGCR, LDL-R and β-actin was assayed by Western blotting in cells treated with sauchinone for 12 hours. (**G**) SREBP2 luciferase activity. The relative luciferase activity was measured in HepG2 cells that had been treated with sauchinone (10, and 100 μM) for 24 hours after transfection with the SREBF2-luciferase construct. (**H**) The mRNA expression of SREBF1, SREBF2, HMGCR and LDL-R were assayed by qRT-PCR in cells treated with sauchinone (20, and 100 μM) and simvastatin (100 μM) for 24 hours. Sau: sauchinone. Statistical significance of the differences between each treatment group and the normal group (*p < 0.05) or the simvastatin- treated groups (^#^*p* < 0.05) was determined. Sau, sauchinone (magnification: ×60, scale bars: 20 µm).

To validate the results of the pathway analysis, we assessed the expression levels of the associated proteins sauchinone treated HepG2 cells. PCSK9 was decreased in sauchinone and simvastatin-treated compared to untreated cells (Fig. 4A,B,C and E). Additionally, PCSK9 was inhibited by sauchinone in mouse primary hepatocytes (Fig. 4D). LDLR is a target of PCSK9, and previous studies have generally reported an inverse relationship with PCSK9^12^. The presently-observed LDLR increase might be due to the down-regulation of PCSK9 expression in sauchinone-treated HepG2 cells (Fig. 4F), not increased LDLR mRNA expression. Additionally, SREBP-1 was also downregulated by sauchinone treatment. However, the combination sauchinone and simvastatin treatment did not appear to decrease SREBP-1 when compared with simvastatin treatment. In contrast, SREBP-2 expression was significantly suppressed by sauchinone treatment or the combination treatment with sauchinone and simvastatin, compared with simvastatin treatment, implying that the combination treatment might potentiate the inhibition of SREBP-2 expression. To more completely comprehend SREBP2 activity in cells treated with sauchinone, SREBF2 reporter gene assays were conducted using HepG2 cells transfected with luciferase-based reporter construct. Thus, sauchinone enabled to prevent SREBP2-mediated PCSK9 activity in hepatocytes (Fig. 4G). Also, SQLE and HMGCR, the downstream targets of SREBP-2 and major enzymes in the cholesterol metabolic pathway, were significantly downregulated by sauchinone-treated HepG2 cells compared with non-treated HepG2 cells (Fig. 4H).

### Sauchinone activates LDLR expression and increases cholesterol uptake

**Figure 5:**
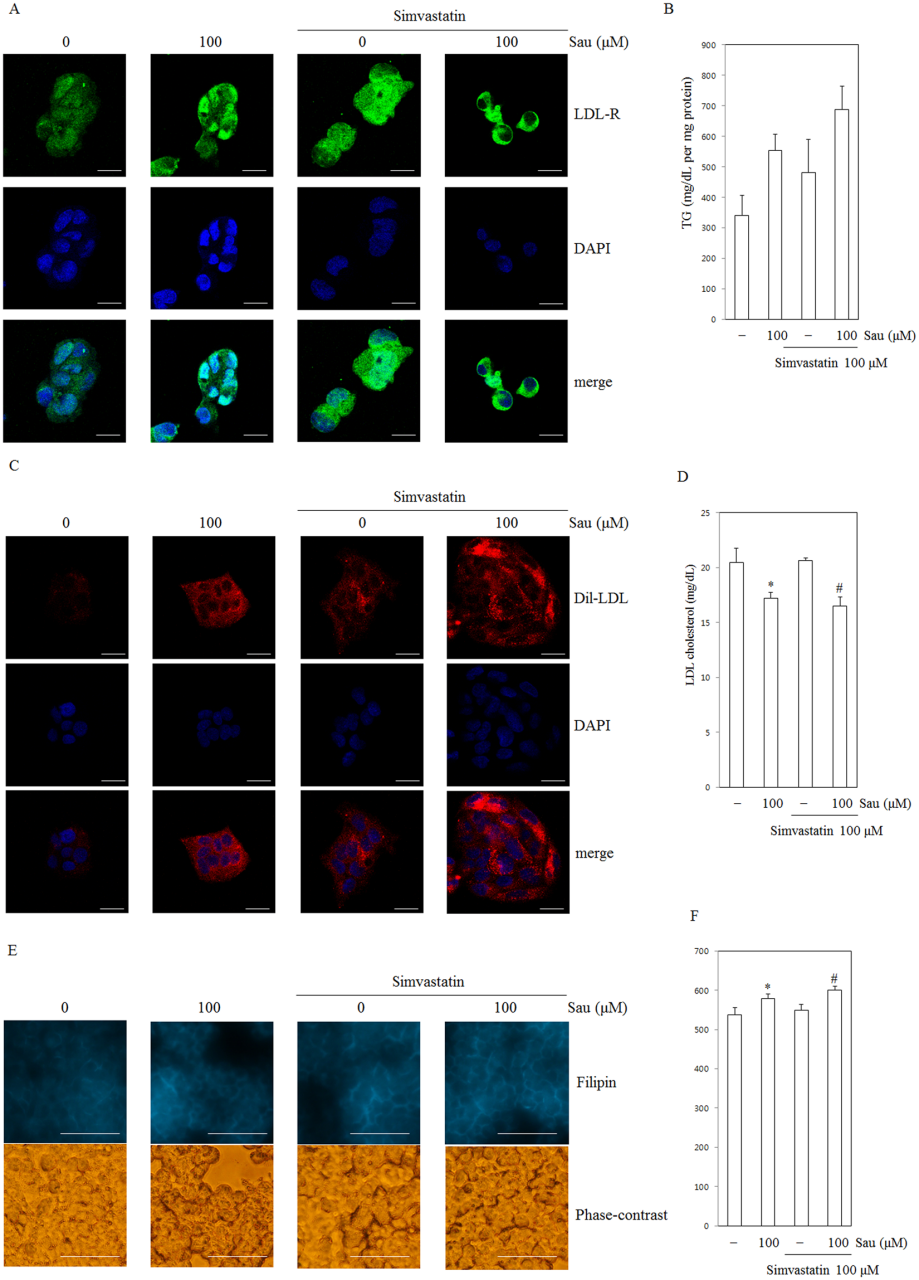
Effect of sauchinone on LDLR expression and cholesterol uptake in the HepG2 human hepatocellular liver carcinoma cell line. (**A**) Expression of LDLR by sauchinone. Cells were cultured for 24 hours, fixed, permeabilized, and incubated with a rabbit monoclonal anti- LDLR antibody followed by an Alexa 488 conjugated anti-rabbit IgG (green). The nuclei of the corresponding cells were visualized by 4′,6-diamidino-2-phenylindole (DAPI) staining (blue). Sau, sauchinone (magnification: ×60, scale bars: 20 µm). (**B**) Quantification of TG in HepG2 cell lysate by ELISA kit (Cell Biolabs, Inc., San Diego, CA, USA) revealed higher uptakes of cholesterol in HepG2 cell lines. (**C**) Effect of sauchinone on intracellular accumulation of 3,3′-dioctadecylindocarbocyanine-low density lipoprotein (DiI-LDL) in HepG2 cells incubated with DiI-LDL (10 μg/mL) under control conditions or treated with 100 μM sauchinone or simvastatin. Representative images are shown. Sau, sauchinone (magnification: ×60, scale bars: 20 µm). (**D**) Quantification of remaining LDL cholesterol in media by ELISA kit (Cell Biolabs, Inc., San Diego, CA, USA) revealed higher uptakes of cholesterol in HepG2 cell lines. (**E**) Filipin staining of HepG2 cells cultured for 24 hours in cells treated with sauchinone (100 μM) and simvastatin (100 μM) as indicated. After 24 hours, cells were washed, fixed in paraformaldehyde and stained with filipin. Intracellular filipin-cholesterol complexes were visualized by fluorescence microscopy and images were captured with a fluorescence microscope. Representative images are shown. Sau, sauchinone (magnification: ×40, scale bars: 50 µm). (**F**) Median fluorescence of filipin-stained cells treated as described in E. Statistical significance of the differences between each treatment group and the normal group (*p < 0.05) or the simvastatin- treated groups (^#^*p* < 0.05) was determined. Sau, sauchinone (magnification: ×60, scale bars: 20 µm).

LDLR is a key cell surface receptor for cholesterol uptake. We tested the effects of sauchinone on LDLR expression in HepG2 cells by immunofluorescence. LDLR markedly increased by 24 hours after treatment with sauchinone. As a consequence of PCSK9 inhibition and elevated LDLR expression, we assessed cellular regulation of cholesterol in HepG2 cells. For the experiments, cells were incubated in medium with 10% lipoprotein-deficient serum (LPDS; Millipore Corporation, Bedford, MA) supplemented with LDL-C (30 µg/mL of cholesterol), and exposed to sauchinone or the DMSO (0.1% control). To determine if sauchinone can improve cholesterol uptake, we monitored intracellular free cholesterol and LDL-C by staining cells with filipin and 1,1′-dioctadecyl-3,3,3,3′-tetramethylindocarbocyanineperchlorate (DiI)-labeled LDL. HepG2 cells were incubated with DiI-LDL for 4 hours in the absence (control) or the presence of sauchinone (100 µM) and then examined by confocal microscopy. As shown in Fig. 5C, treatment with sauchinone caused an increase DiI. Similarly, treatment of cells with sauchinone for 24 hours increased the free cholesterol content of HepG2 cells (Fig. 5E). The quantification of the filipin fluorescence showed a slightly increased signal in HepG2 cell lines (Fig. 5F). Also, the amount of LDL-C remaining in the sauchinone treated cell culture medium was significantly lower than normal cells. (Fig. 5D).

### PCSK9 knockdown inhibits sauchinone regulation of cholesterol metabolism

**Figure 6:**
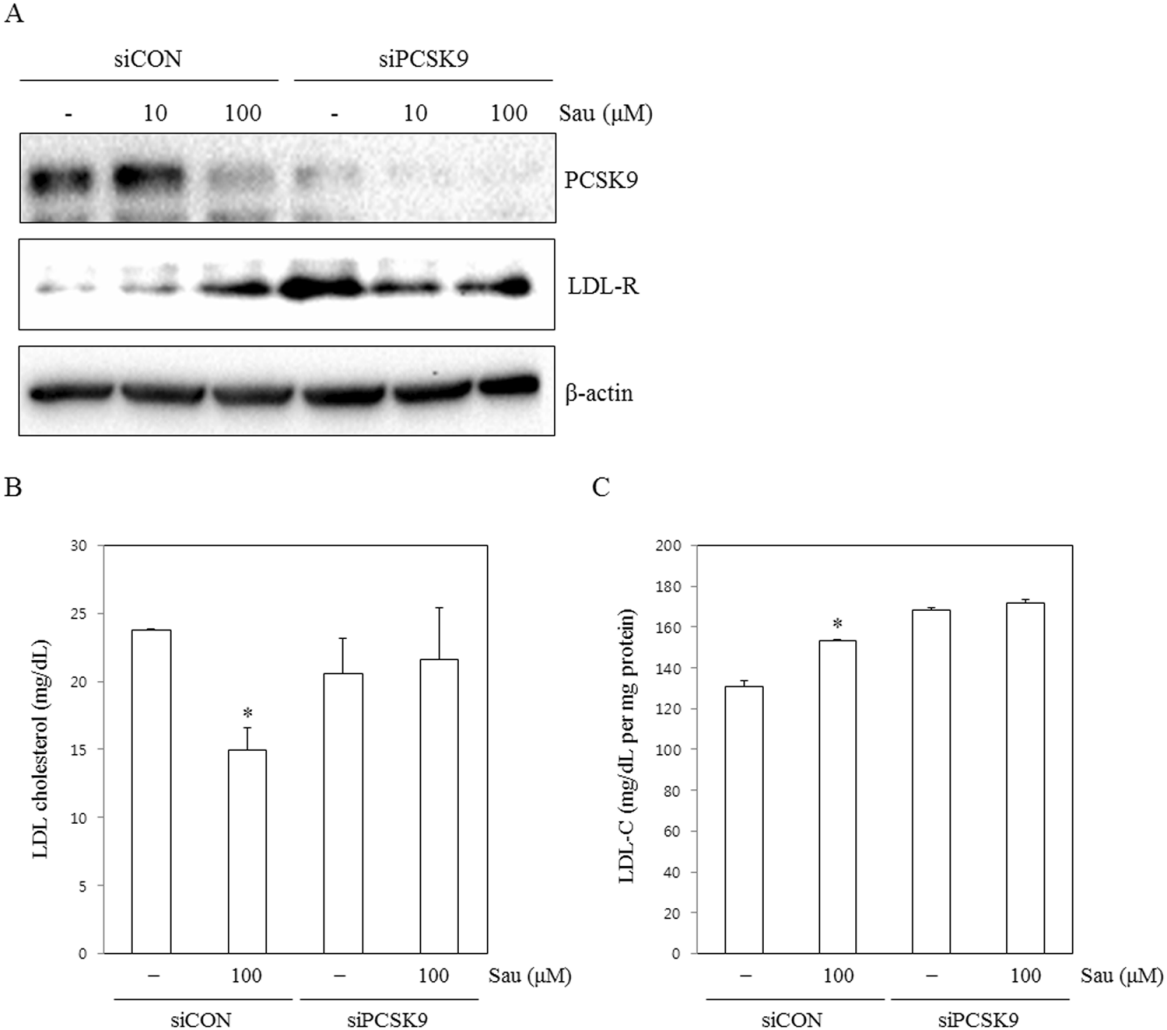
PCSK9 knockdown inhibits the effect of sauchinone. Identification of efficient PCSK9-siRNA sequences and measurement of the relative PCSK9 concentration 48 hours after transfection. HepG2 cells transfected with PCSK9-siRNA for 48 hours were treated with sauchinone (10 and 100 μM) for an additional 24 hours. (**A**) The expression of PCSK9 and LDL-R was assayed by Western blot analysis. (**B**) Quantification of remaining LDL cholesterol in media and (**C**) LDL cholesterol in HepG2 cell lysate by ELISA kit (Cell Biolabs, Inc., San Diego, CA, USA) revealed higher uptakes of cholesterol in HepG2 cell lines. Statistical significance of the differences between each treatment group and the normal group (*p < 0.05).

We also investigated whether PCSK9 can directly regulate cholesterol metabolism in PCSK9 knockdown HepG2 cells in response to sauchinone treatment. The efficacy of PCSK9 silencing by the selected small interfering (si)RNA was confirmed by westernblot. Forty eight hours after transfection, HepG2 cells were treated with sauchinone (50 μM) and LDLR expression was monitored. As expected, the inhibition of total LDLR protein expression by sauchinone was significantly abrogated by PCSK9-siRNA, while the LDLR level was still slightly up-regulated by sauchinone (Fig. 6).

### Sauchinone inhibits hepatic steatosis and lipogenic gene induction in mice

**Figure 7:**
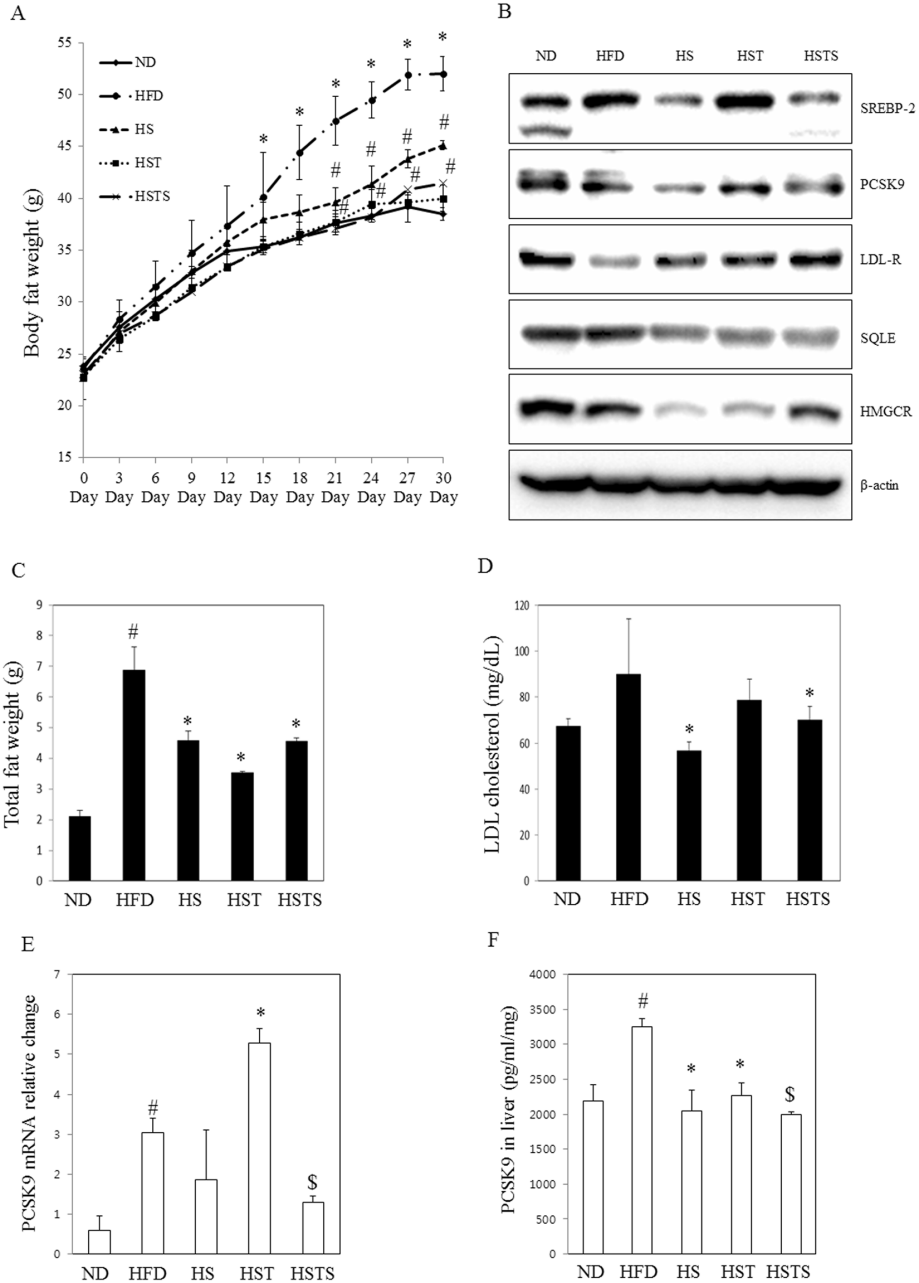
Effects of long-term treatment with sauchinone on the risk of occurrence of hypercholesterolemia in mice. (**A**) Effects of sauchinone on body weight gain. (**B**) The protein expression of SREBP2, PCSK9, LDL-R, SQLE, HMGCR and β-actin was assayed by Western blotting in liver. (**C**) Total fat weight, and (**D**) LDL-cholesterol in plasma in the ND, HFD, HS, HST, and HSTS group. (**E**) PCSK9 mRNA expression was assayed by qRT-PCR in the ND, HFD, HS, HST, and HSTS groups. (**F**) The protein expression of PCSK9 in the liver was assayed by ELISA. Statistical significance of the differences between each treatment group and the ND (^#^*p* < 0.05), HFD (*p < 0.05) or HST (^$^*p* < 0.05) group was determined.

Based on the *in vitro* data, an *in vivo* experiment using high-fat diet (HFD)-fed mice was carried out to investigate the effect of sauchinone on the downregulation of LDL-C. HFD feeding for 4 weeks resulted in a marked increase in body weight gain (Fig. 7A). Treatment of mice with sauchinone (100 mg/kg/day) significantly decreased body weight gain at the last 14 days. However, food intake was unchanged by sauchinone treatment (data not shown). PCSK9 protein levels were reduced in the HFD diet supplemented with 100 mg/kg/day sauchinone (HS), HFD diet supplemented with 20 mg/kg/day simvastatin (HST), and HFD diet supplemented with 100 mg/kg/day sauchinone and 20 mg/kg/day simvastatin (HSTS) groups (Fig. 7B). Consistent with the literature^35^ and *in vitro* data, statin activated the expression of SREBP-2, a transcription factor that activates both the LDLR and PCSK9. SREBP-2 was suppressed by sauchinone or the combination of sauchinone and simvastatin when compared with HFD and HST group. HMGCR and SQLE were also decreased in the HS, HST, and HSTS groups. Increased LDLR expression by treatment with sauchinone or in combination with simvastatin was observed *in vitro* and *in vivo*. Mice fed a HFD for 30 days, exhibited marked increases in total fat, while mice fed the HS, HST, or HSTS diets for 15 days (from day 15 to day 30) showed significantly decreases in all these fats on day 30 (Fig. 7C). With respect to LDL-C, the concentration of LDL-C in the HS and HSTS groups was significantly lower than the HFD group (Fig. 7D). Moreover, analysis of PCSK9 in the liver using immunoblot and qRT-PCR showed that its expression was elevated in the HFD and HST groups, while it was significantly inhibited in the HS and HSTS (Fig. 7E). PCSK9 protein levels were reduced in the HS, HST, and HSTS groups (Fig. 7F).

## Discussion

Cholesterol is generated by dietary uptake through the LDLR and endogenous cholesterol biosynthesis^36^. In normal mammalian cells, cholesterol metabolism is maintained at a steady level through complex regulatory mechanisms. Gene ontology (GO) and KEGG pathway analyses have shown that the top 5 pathways with equally strong prediction scores computed by false discovery rate (FDR) are steroid/cholesterol synthesis and metabolism in response to sauchinone treatment. These data suggest that sauchinone might modulate the cholesterol pathway. Additionally, sauchinone can inhibit intracellular cholesterol levels by inhibiting SQLE, HMGCR, and PCSK9 in HepG2 cells, supporting the involvement of the cholesterol pathway.

PCSK9 is a serine protease that is involved in the regulation of LDLR. LDLR is receptor for serum LDL^37^. PCSK9-induced degradation of LDLR results in reduced intracellular cholesterol uptake and increased serum LDL-C levels^13,38^. Comprehensive gene analysis suggested that the genes encoding the HMGCR, SQLE, 3-hydroxy-3-methylglutaryl-Coenzyme A synthase 1 (HMGCS1), CETP, and PCSK9, which are regulated by SREBP-2, might be responsible for cholesterol lowering effects by sauchinone. PCSK9 knockdown recede the increase in LDLR activity mediated by sauchinone, suggesting that PCSK9 has a key role in cholesterol metabolism and that PCSK9 and LDLR expression are inversely correlated. SREBP-2 activates PCSK9 and LDLR transcription by binding to functional sterol regulatory elements in the promoters of these genes^39^. Various genes including hepatocyte nuclear factor 1 alpha (HNF1A), Baculoviral IAP repeat-containing protein 2 (BIRC2), beta-secretase 1 (BACE1), Annexin A2 (ANXA2), cooperates with SREBF2 to promote PCSK9 transcription^40–43^. PCSK9 inhibitors such as curcumin and berberine, also regulate PCSK9 expression via HNF1A^35,44^. However, we observed no changes of HNF1A, BIRC2, BACE1, and ANXA2 in the DEG analysis in sauchinone treated HepG2 cells (Supplemental Data File 1). Moreover, we observed downregulation of SREBF2 expression in response to sauchinone treatment, suggesting that SREBF2 is the upstream transcription factor that regulates sauchinone prevented expression of PCSK9. Additionally, SREBP-1 was also downregulated by sauchinone treatment. However, the combination treatment with sauchinone and simvastatin did not appear to decrease SREBP-1 when compared with simvastatin treatment. In contrast, SREBP-2 expression was significantly suppressed by sauchinone treatment or the combination treatment with sauchinone and simvastatin when compared with simvastatin treatment, implying that the combination treatment might possess potentiating inhibition on SREBP-2 expression (Fig. 4). Also, SQLE and HMGCR, the downstream targets of SREBP-2 and major enzymes in the cholesterol metabolic pathway, were significantly downregulated by sauchinone-treated HepG2 cells compared with non-treated HepG2 cells. Moreover, in HepG2 cells incubated in the presence of a relatively high concentration of LDL cholesterol, sauchinone increased LDL-receptor mRNA levels, the expression of LDL-receptor protein, and the uptake of DiI-LDL into cells, and thereby intracellular cholesterol levels increased. Also, the elevated TG level was observed (Fig. 5). Administration of cholesterol biosynthesis inhibitors such as statins causes an initial depletion in hepatic cholesterol levels^45^. Consistent with the literature^31^ and *in vitro* data, statin activated the expression of SREBP-2, a transcription factor that activates both the LDLR and PCSK9. The rate of the LDLR protein degradation was also increased as a result of upregulation of PCSK9 levels. Some statins reportedly increases plasma PCSK9, resulting in a partial attenuation of the effects of statins on the LDLR expression^46–48^. SREBP-2 was suppressed by sauchinone or the combination of sauchinone and simvastatin when compared with the HFD and HST groups. Additionally, HMGCR and SQLE were also decreased in the HS, HST, and HSTS groups. Moreover, as the increase in LDLR expression by treatment with sauchinone or in combination with simvastatin *in vitro* was observed, there was significant increase in LDLR *in vivo* (Fig. 7B). From these observations, it was concluded that sauchinone or the combination of sauchinone and simvastatin can reduce LDL-C levels by inhibiting PCSK9 expression, and by increasing LDLR expression, in the *in vivo* model.

The collective results implicate sauchinone as being potentially valuable in the treatment of hepatic steatosis, as it inhibits PCSK9 induction and HFD-induced hepatic steatosis. Furthermore, sauchinone strongly regulates cholesterol metabolism target genes such as SREBP-2, HMGCR, SQLE, and LDLR in liver, adding value to its use for cholesterol homeostasis or cardiovascular diseases.

## Methods

### Cell culture, drugs and chemicals

The HepG2 human hepatocellular liver cell line was obtained from the Korea Research Institute of Bioscience and Biotechnology (South Korea) and grown in Eagle’s Minimum Essential Medium (EMEM) containing 10% fetal bovine serum and 100 U/mL penicillin/streptomycin sulfate. Cells were incubated in a humidified 5% CO_2_ atmosphere at 37 °C. EMEM, penicillin, and streptomycin were purchased from Hyclone (Logan, UT, USA). Bovine serum albumin was purchased from Sigma-Aldrich (St. Louis, MO, USA). DiI-LDL was purchased from invitrogen (Invitrogen, Carlsbad, CA). Antibodies against LSS, SREBP1, SREBP2, PCSK9, HMGCR, LDL-R, SQLE and β-actin were purchased from Abcam, Inc. (Cambridge, MA, USA). PCSK9, LDL-R, HMGCR, SREBP1, SREBP2, HNF1A, BIRC2, ANXA2 and glyceraldehyde-3-phosphate dehydrogenase (GAPDH) oligonucleotide primers were purchased from Bioneer Corp. (Daejeon, South Korea). Sauchinone was isolated from the chloroform fraction of *Saururus chinensis*, as previously described, and confirmed by a spectroscopic analysis^32^. The purity of sauchinone was determined to be over 95% by high performance liquid chromatography using an ultraviolet detector.

### Library preparation and sequencing

Total cellular RNA was isolated using a Trizol RNA extraction kit (Thermo Fisher Scientific, Waltham, MA, USA) according to the manufacturer’s instructions. The quantity and quality of the total RNA were evaluated using a model Agilent 2100 bioanalyzer RNA kit (Agilent Technologies, Santa Clara, CA, USA). The isolated total RNA was processed for preparing mRNA sequencing library using a TruSeq Stranded mRNA Sample Preparation kit (Illumina, San Diego, CA, USA) according to the manufacturer’s protocol. Quality and size of libraries were assessed using Agilent 2100 bioanalyzer DNA kit (Agilent Technologies). All libraries were quantified by qPCR using CFX96 Real Time System (Bio-Rad Hercules, CA, USA) and sequenced on the NextSeq 500 sequencers (Illumina) with a paired-end 75 bp plus single 6 bp index read run. All data have been deposited in the NCBI Gene Expression Omnibus (GEO) public repository and can be accessed through the accession number GSE113247.

### Preprocessing and genome mapping

To assess the quality and soundness of the raw RNA sequence reads, we evaluated base quality distribution and inclusion of the adapter sequences of the raw reads using FastQC (http://www.bioinformatics.babraham.ac.uk/projects/fastqc) software.

Potentially existing sequencing adapters and raw quality bases in the raw reads were trimmed by cutadapt software^49^. The option -a AGATCGGAAGAGCACACGTCTGAACTCCAGTCAC and -A AGATCGGAAGAGCGTCGTGTAGGGAAAGAGTGTAGATCTCGGTGGTCGCCGTATCATT were used for the common adapter sequence of the Illumina TruSeq adapters and the option −q 0, −m 20, −O 3 was used for trimming low quality 5′ and 3′ ends of the raw reads. The cleaned high quality reads after trimming the low quality bases and sequencing adapters were mapped to the mouse reference genome mm10 of the UCSC genome (https://genome.ucsc.edu) by STAR software^50^. Since the sequencing libraries were prepared strand-specifically by using Illumina’s strand-specific library preparation kit, the strand-specific library option,–library-type fr-firststrand was applied in the mapping process.

### Quantifying gene expression and differential expressed gene analysis

Cufflinks software was used to quantify the mapped reads on the mouse reference genome in to the gene expression values^51^. The gene annotation of the mouse reference genome mm10 from UCSC genome (https://genome.ucsc.edu) in GTF format was used as the gene model and the expression values were calculated in Fragments Per Kilobase of transcript per Million fragments mapped (FPKM) unit. The differentially expressed genes between the two selected biological conditions were analyzed by Cuffdiff software in Cufflinks package^51^. To compare the expression profiles among the samples, the normalized expression values of the few hundred selected genes with the most variable expression were unsupervised clustered using MeV software of the TM4 microarray software suite^52^. The scatter plots for the gene expression values and the volcano plots for the expression-fold changes and p-values between the two selected samples were drawn by in-house R scripts.

### Functional category analysis

The biological functional role of the differential gene expression between the compared biological conditions was assessed using the gene set overlapping test between the analyzed differential expressed genes and functional categorized genes, including biological processes of Gene Ontology (GO), KEGG pathways, and transcription factor binding target gene sets by the DAVID tool^53^.

### Assay of PCSK9 Secretion

Secreted human and mouse PCSK9 level in culture supernatants of cells were measured using an enzyme-linked immunosorbent assay (ELISA) kits according to the manufacturer’s instructions (BioVision, CA, USA; Elabscience Biotechnology, Wuhan, China). Color development at 450 nm was then measured using an automated microplate ELISA reader. In addition, a standard curve was generated for each assay plate by measuring the absorbance of serial dilutions of recombinant human and mouse PCSK9 at 450 nm.

### Animals

All procedures and the experimental protocol were approved by the Institutional Animal Care and Use Committee of Dongguk University-Seoul (#2012-0673; IACUC-2015-064) and were performed in accordance with relevant guidelines and regulations. Male ICR and C57BL/6 mice obtained from Charles River Orient (Seoul, South Korea) were acclimated for 1 week prior to the start of the study. Upon arrival, the animals were randomized and housed at three per cage under strictly controlled environmental conditions (20–25 °C and 48–52% relative humidity). A 12-hour light/dark cycle was used at an intensity of 150–300 lux. The ICR mice were started on a 30-day normal diet (ND) in which 10% of the kilocalories were supplied as fat (Product N12450B; Research Diets, New Brunswick, NJ, USA) or a high-fat diet (HFD) in which 60% of the kilocalories were supplied as fat (Product D12592; Research Diets), for 30 days. After 1 week (on day 7) mice in the ND and HFD groups were distributed into five treatment groups, with eight mice in each group. The mice fed with a HFD were orally administered with either simvastatin (20 mg/kg/day; HST group) or 100 mg/kg/day sauchinone (HS group) or combination of simvastatin and sauchinone (20 mg/kg/day and 100 mg/kg/day, respectively, HSTS group) dissolved in polyethylene glycol (PEG) 400 and distilled water (1:9, v/v) every day during the last 15 days of feeding.

### Preparation and stimulation of mouse primary hepatocytes

Mouse primary hepatocytes were prepared from 8 weeks-old C57BL/6 male mice livers. Hepatocytes were isolated from mice by non-recirculating collagenase perfusion through the portal vein as previously described^54^. The isolated mouse hepatocytes were plated on dishes coated with rat collagen type I, confluence after plating was 80–90%, with hepatocyte viability of greater than 90% as assessed by Trypan blue exclusion. After plating, hepatocytes were cultured in William’s Hepatocyte medium (Gibco, NY, USA) containing 10% FBS at 37 °C in 5% CO_2_ atmosphere.

### Hematoxylin and Eosin Staining in the liver

The left lateral lobe of the liver was excised, fixed in 10% formalin, and embedded in paraffin. Four micrometer thick sections of liver was obtained and stained with hematoxylin and eosin (H&E) for the histological examination of adipocytes, as previously described^55^.

### Liver and fats weights and plasma analysis

At the end of the experimental period, the liver and total fat was extracted and the weights of the components were measured. In the case of fat, the weights of total, epididymal, inguinal, and retroperitoneal fats were measured separately. The plasma low-density lipoprotein cholesterol (LDL-C), TG and total cholesterol (TC) contents were determined using an ELISA kit (Cell Biolabs, Inc., San Diego, CA, USA).

### Immunofluorescence

HepG2 cells cultured on Permanox plastic chamber slides were fixed with ethanol for 30 min at 4 °C. Following washing with phosphate buffered saline (PBS) and blocking with 3% bovine serum albumin in PBS for 30 min, samples were incubated overnight at 4 °C with rabbit monoclonal anti-PCSK9 or anti-LDLR (1:500 dilution, Abcam, Cambridge, MA, USA). The excess primary antibody was removed, slides were washed with PBS, and the samples were incubated with an Alexa 488-conjugated secondary antibody (Invitrogen Molecular Probes, Burlington, ON, Canada) for 2 h at room temperature. Following washing with PBS, slides were mounted using ProLong Gold Antifade reagent containing 4′,6-diamidino-2-phenylindole (DAPI; Thermo Scientific, Waltham, MA, USA) to visualize the nuclei. Specimens were covered with coverslips and evaluated under a confocal laser scanning microscope (Nikon Eclipse, Nikon, Japan).

### Filipin staining

For Filipin staining, cells grown on coverslips were fixed with 4% paraformaldehyde for 30 min at room temperature, followed by 2 hrs incubation in a freshly prepared Filipin III (Sigma-Aldrich, St. Louis, MO, USA) solution (50 μg/mL). To make fresh Filipin III solution, Filipin III was dissolved in 10 μL of DMSO first, then diluted with 200 μL of PBS, and was used immediately. Specimens were covered with coverslips and evaluated under a fluorescence microscope (Nikon Eclipse, Nikon, Japan).

### Immunoblot analysis

Protein expression was assessed by Western blotting according to standard procedures. Briefly, HepG2 cells were cultured in 60-mm culture dishes (2 × 10^6^/mL), following by pretreatment various concentrations of sauchinone (20 and 100 μM). Cells were washed twice in ice cold PBS (pH 7.4), the cell pellets were resuspended in lysis buffer on ice for 15 min, and the cell debris was then removed by centrifugation. Protein concentration was determined using protein assay reagent (Bio-Rad) according to the manufacturer’s instructions. Protein (20–30 μg) was mixed 1:1 with 2 × sample buffer (20% glycerol, 4% SDS, 10% 2-ME, 0.05% bromophenol blue, and 1.25 M Tris [pH 6.8]), loaded onto 8 or 15% SDS-polyacrylamide gel electrophoresis gels, and run at 150 V for 90 min. Cellular proteins were transferred onto ImmunoBlot polyvinylidene difluoride membranes (Bio-Rad) using a Bio-Rad semi-dry transfer system according to the manufacturer’s instructions. The membranes were then incubated overnight with the resprective primary antibody (diluted 1:500–1:1000) in Tris-buffered saline containing 5% skimmilk and 0.1% Tween 20. The following day, the blots were washed three times with Tris-buffered saline (0.1% Tween 20) and incubated for 1 h with an horseradish peroxidase conjugated secondary anti-IgG antibody (diluted 1:2000–1:20,000). The blots were washed again three times with Tris-buffered saline (0.1% Tween 20), and immunoreactive bands were developed using the chemiluminescent substrate ECL Plus (Amersham Biosciences, Piscataway, NJ, USA). Images were acquired by using a ChemiDoc Imaging system (ChemiDoc™ XRS system with Image Lab™ software 3.0; Bio-Rad, Hercules, CA, USA).

### Quantitative real-time RT-PCR

Total cellular RNA was isolated using a Trizol RNA extraction kit according to the manufacturer’s instructions. Briefly, total RNA (1 μg) was converted to cDNA by treatment with 200 units reverse transcriptase and 500 ng oligo-dT primers in 50 mM Tris-HCl (pH 8.3), 75 mM KCl, 3 mM MgCl_2_, 10 mM dithiothreitol, and 1 mM dNTPs at 42 °C for 1 h. The reaction was stopped by incubating the solution at 70 °C for 15 min, after which 1 µL cDNA mixture was used for enzymatic amplification. PCR reactions were performed using 1 μL cDNA and 9 μL master mix containing iQ SYBR Green Supermix (Bio-Rad), 5 pmol of forward primer, and 5 pmol reverse primer, in a CFX384 Real-Time PCR Detection System (Bio-Rad). The reaction conditions were 3 min at 95 °C followed by 40 cycles of 10 s at 95 °C and 30 s at 55 °C. The plate was subsequently read. The fluorescence signal generated with SYBR Green I DNA dye was measured during the annealing steps. The specificity of the amplification was confirmed using a melting curve analysis. Data were collected and recorded by CFX Manager Software (Bio-Rad) and expressed as a function of the threshold cycle (C_T_). The relative quantity of the gene of interest was then normalized to the relative quantity of GAPDH (ΔΔC_T_). The mRNA abundance in the sample was calculated using the equation 2^−(ΔΔCT)^. The following specific primer sets were used (5′ to 3′): human - GAPDH: GAAGGTGAAGGTCGGAGTCA (forward),AATGAAGGGGTCATTGATGG (reverse); human - LDLR: GTGCTCCTCGTCTTCCTTCTTTG (forward), TAGCTGTAGCCGCCTGTT (reverse); human - SREBF-1: GGAGGATGGACTGACTTCCA (forward),GGCCTTTCACAGAACAGGAA (reverse); human - SREBF-2: ACCACGCAGAGCACCAAG (forward), GGGAGGAGAGGAAGGAGAGG (reverse); human - HMGCR: TGATTGACCTTTCCAGAGCAAG (forward), CTAAAATTGCCATTCCACGAGC (reverse); human - PCSK9: GGTACTGACCCCCAACCTG (forward), CCGAGTGTGCTGACCATACA (reverse); mouse - Gapdh: TGTTCCTACCCCCAATGTGT (forward), GGTCCTCAGTGTAGCCCAAG (reverse); mouse - Pcsk9: GGCAGAGGCTGATCCACTT (forward), ACCACACCGTCCTACAGAGC (reverse). Gene-specific primers were custom-synthesized by Bioneer.

### Transient transfection and reporter gene assays

Cells were transiently transfected with promoter clone for gene SREBF2 (GeneCopoeia, Rockville, MD, USA) for 24 h in the presence of Lipofectamine 2000 reagent. The activity of luciferase was measured by using Secrete-Pair Dual Luminescence Assay Kit (GeneCopoeia, Rockville, MD, USA) and a luminometer.

### Cell transfection

Gene silencing was performed by transfection LM3 cells with siRNA oligonucleotides (Santa Cruz Biotechnology, Santa Cruz, CA, USA). For transfections in 12-well plates, 1.0 × 10^5^ cells were seeded well and the cells transfected using the Lipofectamine 2000 Transfection Agent (Thermo Fisher Scientific) according to the manufacturer’s protocol.

### Statistical analysis

The data are presented as the mean ± S.E.M. The level of statistical significance was determined by analysis of variance (ANOVA) followed by Dunnett’s t-test for multiple comparisons. P values less than 0.05 were considered to be significant.

## Electronic supplementary material

Supplementary data

Supplemental Tables
