# Supplementary material for: Sauchinone controls hepatic cholesterol homeostasis by the negative regulation of PCSK9 transcriptional network
Source: Sci Rep. 2018 Apr 30;8:6737. doi: 10.1038/s41598-018-24935-6 (PMC5928089; doi:10.1038/s41598-018-24935-6)

**Supplementary data**

**Sauchinone controls hepatic cholesterol homeostasis by the negative regulation of PCSK9 transcriptional network**

Hee-Sung Chae^1^, Byoung Hoon You^1^, Dong-Yeop Kim^2^, Hankyu Lee^1^, Hyuk Wan Ko^1^, Young Hee Choi^1^, Sun Shim Choi^2^, Young-Won Chin^1^

^1^College of Pharmacy and Integrated Research Institute for Drug Development, Dongguk University-Seoul, 32, Dongguk-lo, Ilsandong-gu, Goyang-si, Gyeonggi-do 10326, Republic of Korea

^2^Division of Biomedical Convergence, College of Biomedical Science, and Institute of Bioscience & Biotechnology, Kangwon National University, Chuncheon 24341, South Korea

**Figure S1.** Effects of long-term treatment with sauchinone on the risk of occurrence of hypercholesterolemia in mice. Histologic analysis of liver from normal diet (ND) or high-fat diet (HFD)-fed mice supplemented with sauchinone (HS) or simvastatin (HST) or both (HSTS) for four weeks. Representative photo of gross morphology (left panel) and images of H&E staining from sectioned liver tissue (right panel) indicate that treatment of sauchinone alone or with simvastatin prevent the liver steatosis in HFD-fed mice. (scale bars: 200 µm).

**
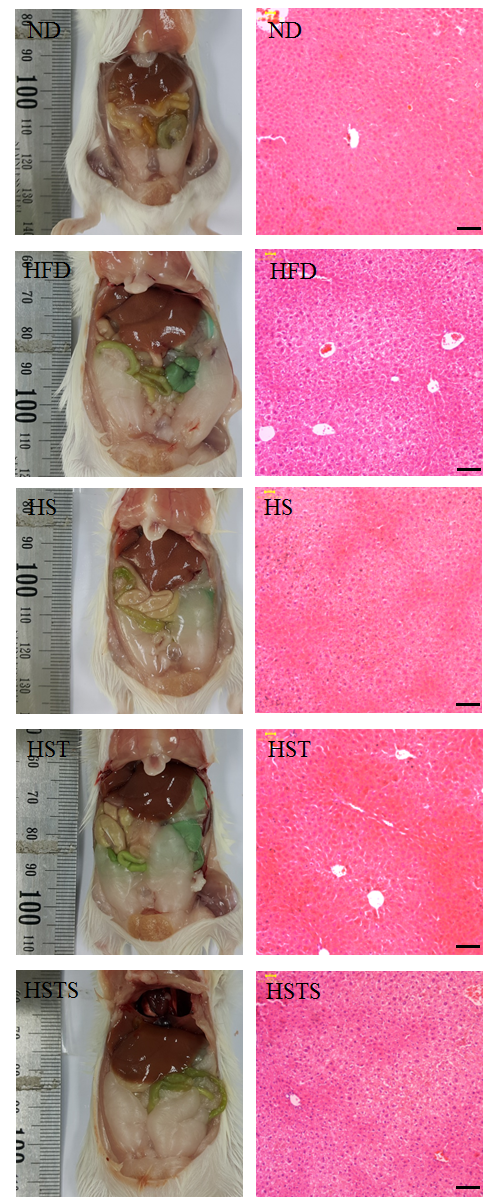
**

**Figure S2.** Modulation of the cholesterol signaling pathway in HepG2 cells treated with sauchinone. (Full length blot of Figure 3B)


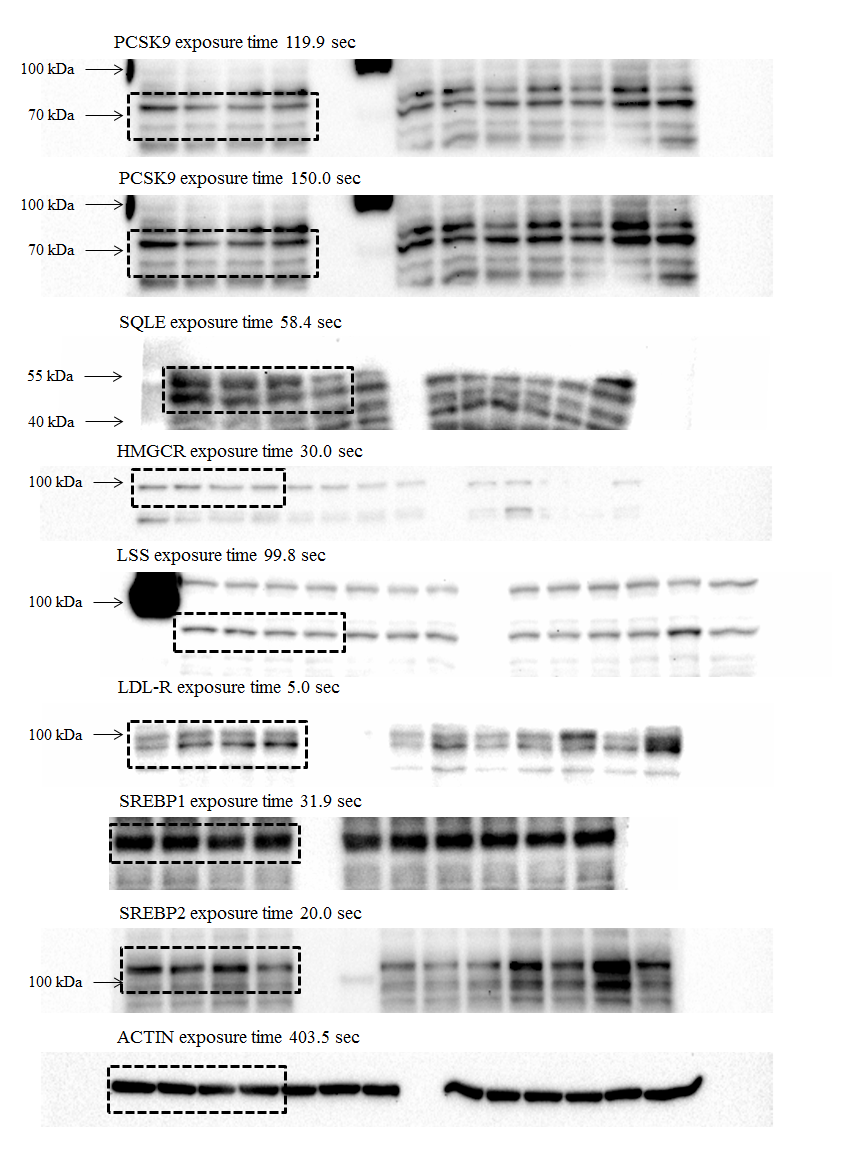


**Figure S3.** Effect of sauchinone on PCSK9 inhibition in the HepG2 human hepatocellular liver carcinoma cell line. (Full length blot of Figure 4B)


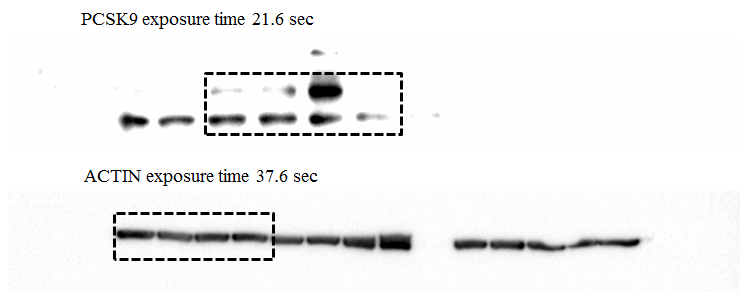


**Figure S4.** Effect of sauchinone on PCSK9 inhibition in the HepG2 human hepatocellular liver carcinoma cell line. (Full length blot of Figure 4D)


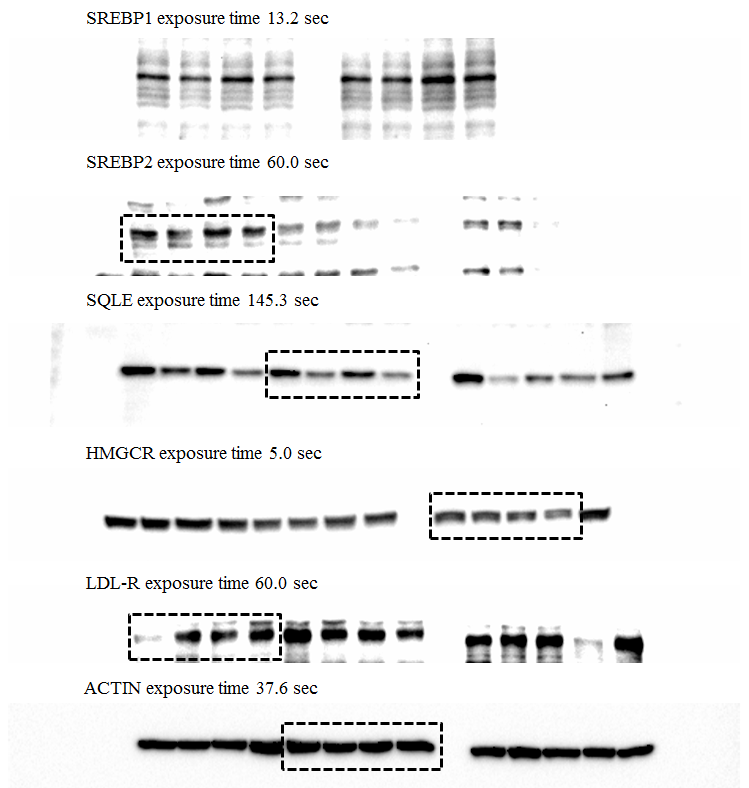


**Figure S5** PCSK9 knockdown inhibits the effect of sauchinone. (Full length blot of Figure 5)


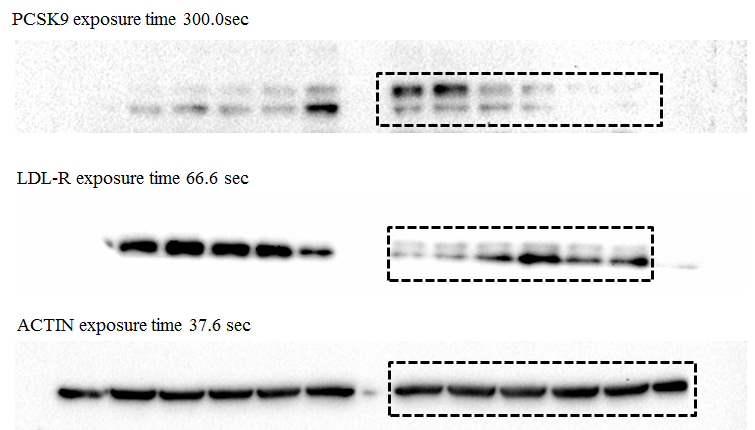


**Figure S6** Effects of long-term treatment with sauchinone on the risk of occurrence of hypercholesterolemia in mice. (Full length blot of Figure 6B)


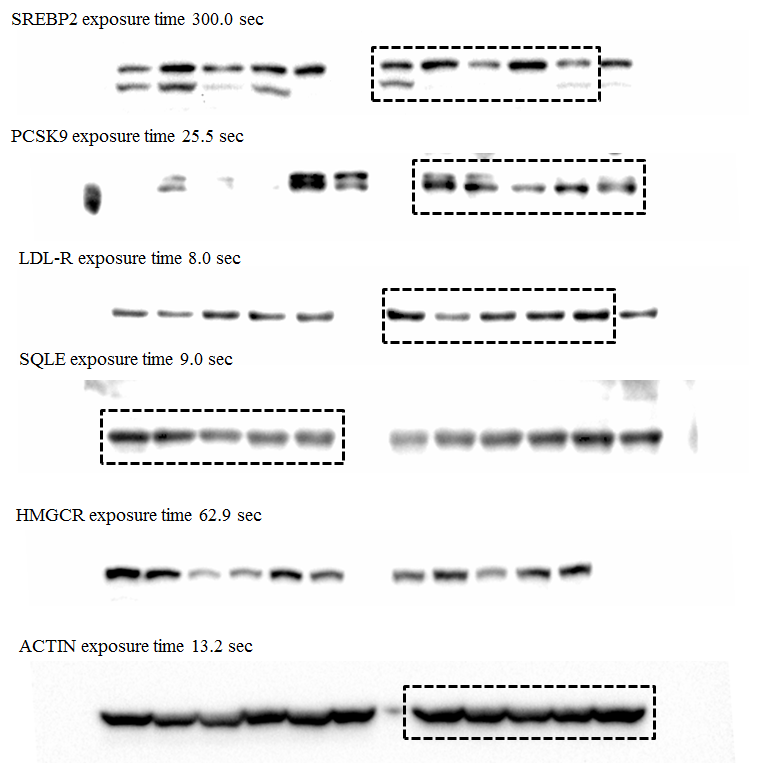


**Figure S7.** Quantitation of Western blots. (**a**) Relative fold changes of Western blot data from figure 3. (b) Relative fold changes of Western blot data from figure 4. (**c**) Relative fold changes of Western blot data from figure 6. (**d**) Relative fold changes of Western blot data from figure 7.

A


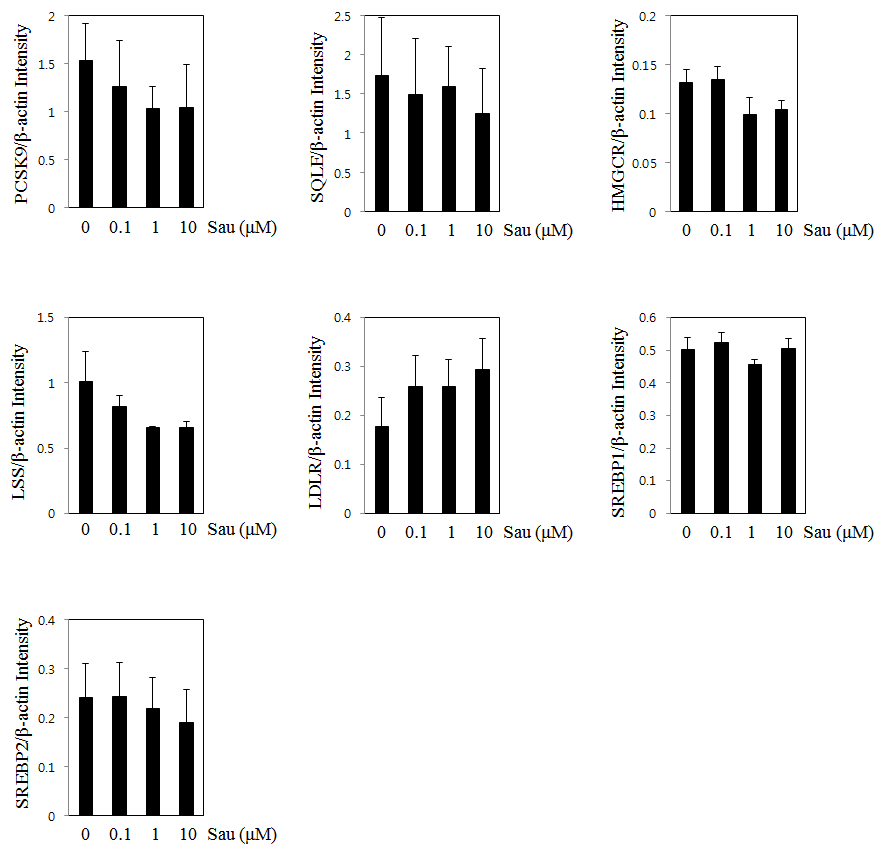


B


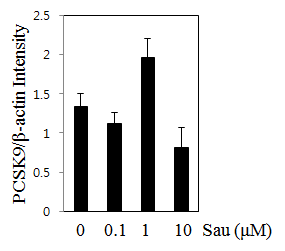


C


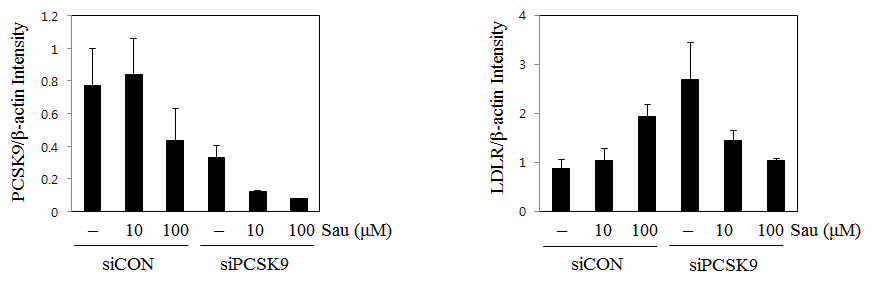


D


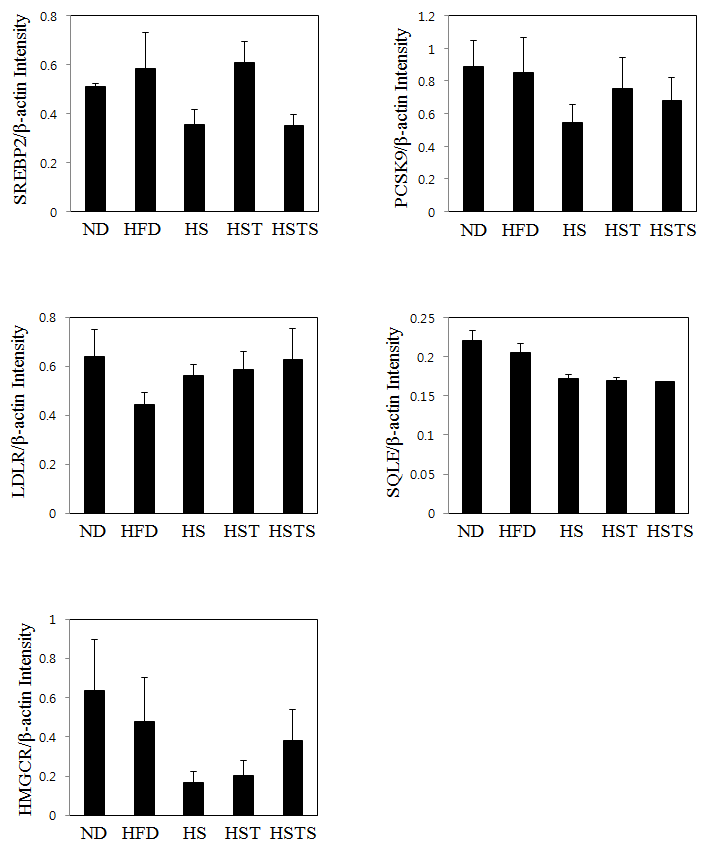

Supplement: Supplementary file 1 — Supplementary data [file 41598_2018_24935_MOESM1_ESM.doc]
